# Perioperative cefazolin prescribing rates following suppression of alerts for non-IgE-mediated penicillin allergies

**DOI:** 10.1017/ash.2023.369

**Published:** 2023-09-29

**Authors:** Ashley Bogus, Kelley McGinnis, Sara May, Erica Stohs, Trevor Van Schooneveld, Scott Bergman

## Abstract

**Background:** Cefazolin is the preferred antimicrobial for prevention of surgical-site infections in most procedures at our institution. Our first alternative is vancomycin which is associated with higher adverse events and infection rates. The presence of penicillin allergies can influence prescribing of vancomycin despite a low risk of cross-reactivity between penicillin and cephalosporins. Nebraska Medicine implemented a systemwide change in April 2022 that suppressed alerts for non–IgE-mediated penicillin allergies in the electronic medical record (EMR, Epic Systems) upon cephalosporin prescribing. We evaluated changes in perioperative antimicrobial surgical infection prophylaxis after this change. **Methods:** We conducted a quasi-experimental study of all patients undergoing procedures for which cefazolin is considered preferred per institutional guidance. Preintervention data were from April 1, 2021, to March 31, 2022, and postintervention data included patients from April 11, 2022, to October 31, 2022, after guidance was distributed to surgeons, operating room staff, and pharmacists. Patients were excluded if they were aged <19 years, had a hospital length of stay <24 hours, underwent procedures after their first throughout the time frame, or received both vancomycin and cefazolin. Statistical significance was set at *P* < .05, determined using the Fisher exact test. **Results:** The study included 6,676 patients: 4,147 in the preintervention group and 2,529 in the postintervention group. We identified 15 procedure categories, with no significant differences between periods (Table 1). The average age was 61 years. Penicillin allergy was reported in 508 patients (12.3%) in the preintervention group and in 319 patients (12.6%) in the postintervention group. In individuals with penicillin allergy, cefazolin prescribing increased from 49.6% to 74.3% (*P* < .01) and vancomycin prescribing decreased from 50.4% to 25.7% (*P* < .01). The largest changes occurred in patients undergoing cardiac, spinal, neurological, and vascular procedures. For patients without penicillin allergy, prescribing remained unchanged. Overall, cefazolin prescribing increased from 92.0% to 95.0% (*P* < .01), and the rate of vancomycin prescribing decreased from 8.0% to 5.0% (*P* < .01) in procedures for which cefazolin was preferred. **Conclusions:** Following the suppression of EMR alerts for non–IgE-mediated allergies when ordering cephalosporins, penicillin prescribing rates of cefazolin for surgical infection prophylaxis improved significantly in procedures for which it was the preferred agent. Further research on infection rates and adverse events with these and other alternative agents are needed.

**Figure:**
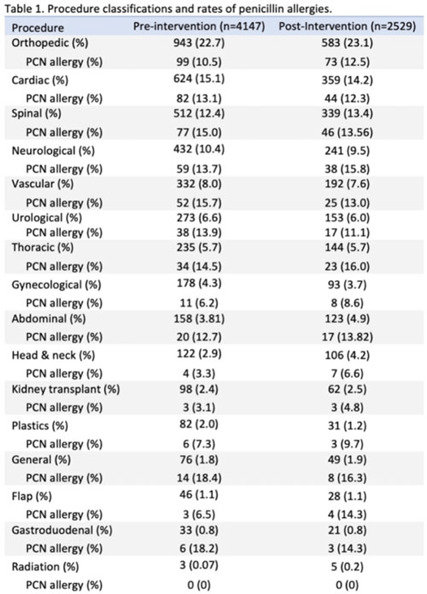

**Figure:**
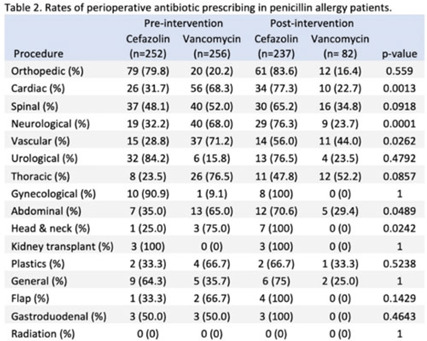

**Disclosures:** None

